# Non-surgical treatment of residual periodontal pockets using sodium hypochlorite/amino acid gel and cross-linked hyaluronic acid—a 9-month pilot randomized controlled clinical trial

**DOI:** 10.1007/s00784-024-05906-w

**Published:** 2024-09-05

**Authors:** Laura Benyei, Anton Friedmann, Thomas Ostermann, Daniel Diehl

**Affiliations:** 1 Department of Periodontology, Faculty of Health, Witten/Herdecke University, 58445 Witten, Germany; 2 Implantat Competence Centrum München, 80333 Munich, Germany; 3 Department of Psychology and Psychotherapy, Faculty of Health, Witten/Herdecke University, 58445 Witten, Germany

**Keywords:** Periodontitis, Cross-linked hyaluronic acid, Adjunctive treatment, Subgingival instrumentation, Sodium hypochlorite/amino acid, Persistent periodontal pockets, Non-surgical periodontal treatment

## Abstract

**Objectives:**

This pilot randomized controlled clinical trial compares the clinical outcome obtained in persistent periodontal pockets during 9-month follow-up of supportive periodontal step 4 treatment performed by either combining subgingival instrumentation with adjunctively used sodium hypochlorite/amino acid gel and crosslinked hyaluronic acid (xHyA) or subgingival instrumentation alone.

**Materials and methods:**

Study protocol is registered under NCT06438354 at Clinicaltrials.gov. Patients seeking further therapy after completed step 2 non-surgical periodontal treatment underwent either repeated subgingival instrumentation with adjunctive application of sodium hypochlorite/amino acid gel and crosslinked hyaluronic acid (group A) or repeated subgingival instrumentation alone (group B). One calibrated investigator performed the treatment sequence in both groups accordingly. Subgingival instrumentation of the residual pockets was carried out under local anaesthesia using hand- and ultrasonic instruments, as well as air polishing in both groups. Patients were instructed to continue oral hygiene without any restriction. At 3-month re-evaluation treatment was repeated accordingly at sites with persistent 5 mm probing depth and BoP + . Clinical attachment level (CAL), pocket probing depth (PPD), gingival recession (GR), and bleeding on probing (BoP) were recorded at baseline (T1), 3- (T2) and 9-month (T3) post-op, with CAL as a primary outcome measure.

**Results:**

In total 52 patients (20 females and 32 males, mean age 58.4 ± 2.4 years) presenting with 1448 sites which required further periodontal treatment were enrolled. Both groups exhibited homogeneity in terms of age, gender, smoking habit, initial number of sites, and BOP. At 9-month evaluation, PD reduction and CAL gain showed significant differences between the test and control group, favouring the adjunctive treatment. GR tended to exhibit more recovery in the test group compared to the control group. Although BOP frequency effectively reduced in both groups, there was no statistically significant difference between the two groups.

**Conclusion:**

Within the limits of the study, the present data indicates that, during subgingival instrumentation of persistent pockets, the adjunctive usage of sodium hypochlorite/amino acid gel and xHyA sufficiently improves the clinical outcomes. The continuous improvement of CAL in association with the GR scores observed in group A, indicates that sites subjected to adjunctive treatment may indicate a tendency for a regenerative response to treatment within the 9-month follow-up period.

## Introduction

Periodontitis, a predominantly chronic inflammatory disease caused by dysbiotic dental plaque biofilms, poses a substantial global health burden. According to the Global Burden of Disease Study, periodontitis ranks sixth worldwide in terms of prevalence, with an alarming 743 million reported cases [1]. Given the progressively aging global population and the increased retention of natural teeth, a heightened global burden is anticipated in the future [2]. Additionally, periodontitis demonstrates bidirectional links with systemic conditions such as atherosclerosis and diabetes [3, 4]. Consequently, the prevention, diagnosis, and effective treatment of periodontitis are paramount.

The primary goal of periodontitis therapy is to resolve periodontal inflammation, expressed by the absence of bleeding, and reduce probing depths to less than 4 mm, preferably alongside with minimal gingival recession [5, 6]. While the efficacy of non-surgical periodontal therapy is evident, residual periodontal pockets are regularly observed at re-evaluation performed during supportive periodontal therapy (SPT). Residual periodontal pockets act as reservoirs for pathogenic bacteria, which lead to the persistence in inflammation and correlate with an increased risk for tooth loss [7]. Since persisting inflammation exhibits a severe risk of disease progression, the timely treatment of respective periodontal pockets is a clinical imperative to establish long-term periodontal stability. Residual or persisting pockets are classified as such if sites present with 4 mm PPD and positive BOP or 5 and more mm in depth. According to the EFP treatment guidelines, these sites are supposed to refer to Step 3 therapy, which may encompass different surgical approaches or repeated instrumentation [8]. However, periodontal surgery may be technically challenging for general practitioners and associated with increased patient morbidity, albeit it remains the most effective treatment option according to the clinical literature in deeper sites [9]. Possible reasons for the inferiority of non-surgical re-instrumentation may include challenging removal of calculus due to restricted accessibility of sites with deep probing depth, or involvement of multi-rooted teeth [10, 11]. For this reason, various adjunctive protocols have been proposed to enhance the efficacy of non-surgical periodontal therapy recently. To date, many adjunctive interventions oscillate between antibacterial effect of local antibiotic chemotherapy, anti-plaque chemical agents, or photodynamic therapy [12, 13]. While the use of biologics, such as enamel matrix derivatives, hyaluronic acid, and platelet-rich fibrin for regenerative periodontal surgery has steadily increased, applying these materials to non-surgical therapy is still uncommon [14–16]. Intriguingly, the integrative application of a sodium hypochlorite/amino acid and crosslinked hyaluronic acid (xHyA) as a regenerative adjunct in the non-surgical treatment of residual periodontal pockets has demonstrated significant attachment level gain in two retrospective studies [17, 18]. However, adequately controlled data supporting the efficacy of this protocol during supportive periodontal therapy is lacking.

This pilot randomized controlled clinical trial investigated the efficacy of adjunctive sodium hypochlorite, and xHyA in treating residual periodontal pockets during supportive periodontal therapy.

## Materials and methods

### Study design

The randomized controlled trial protocol received approval by the Ethics Committee at Witten/Herdecke University (No. 64/2022). The participants informed about the freedom to quit the study whenever they want in agreement with the Helsinki declaration of medical research and confirmed that the data may be used for scientific research. Clinicaltrials.gov registered the study with the ID number: NCT06438354 as a pilot RCT. All participants had been diagnosed with residual periodontal pockets either exceeding 5 mm or equalling 4-5 mm in PPD with bleeding on probing (BOP) at the first re-evaluation after step 2 periodontal therapy [19]. Before enrolment, all patients signed a written informed consent form. After signing the consent form, patients were randomly allocated to either test group (group A, subgingival instrumentation plus adjunctive protocol) or control group (group B, subgingival instrumentation only).

The study was conducted according to current standards of clinical research (CONSORT guidelines: http://www.consort-statement.org/) [20] between August 2022 and October 2023 in a private dental office in Munich, Germany.

### Inclusion and exclusion criteria

**Fig. 1:**
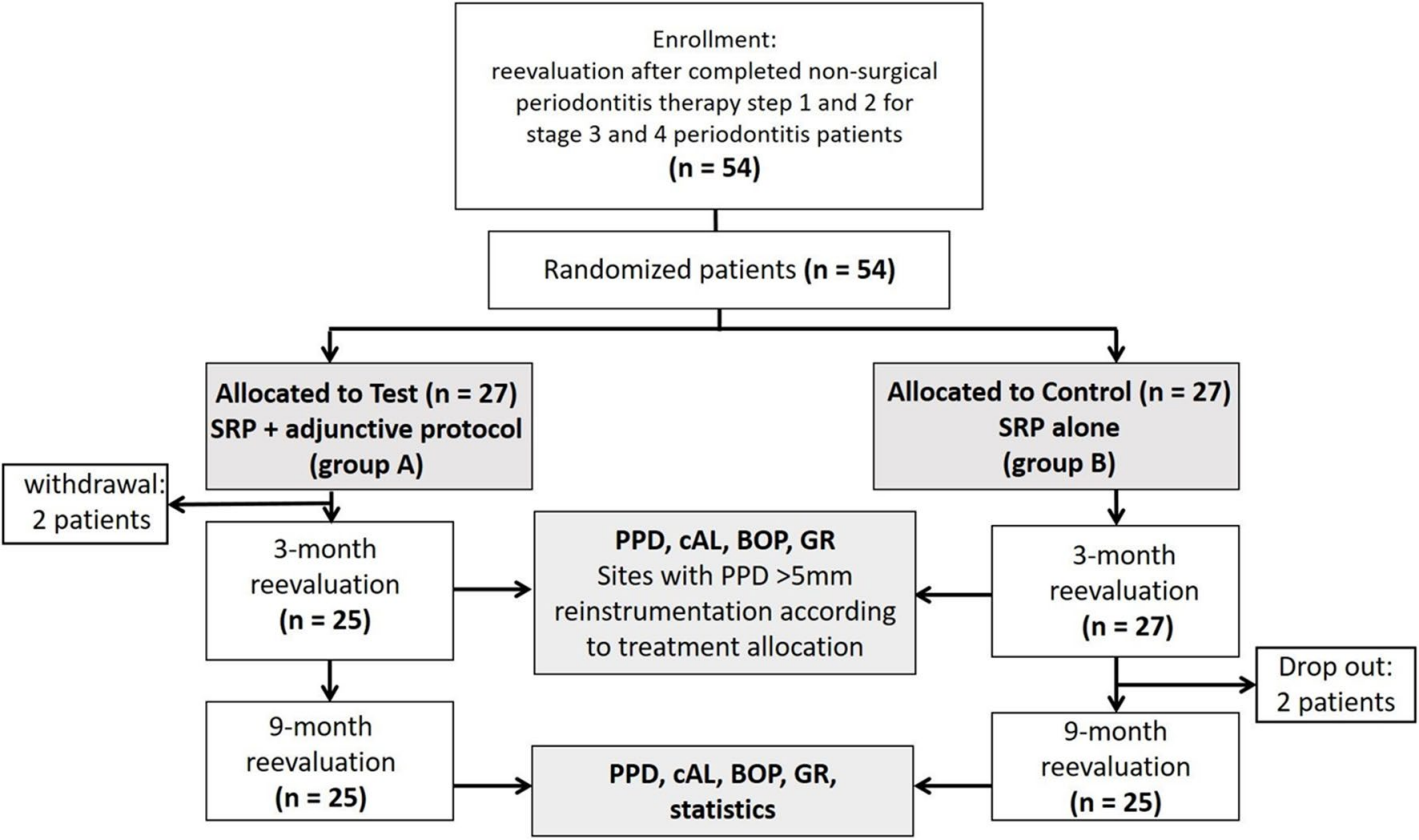
The study outline

The inclusion criteria encompassed systemically healthy adult individuals who were previously diagnosed with periodontitis according to the clinical practice guideline and who had completed step 1 and 2 therapy initially presenting with untreated periodontitis at stage 3 or 4 (Fig. 1) [8]. In particular, the study focused on persistent periodontal pockets characterized by a probing pocket depth (PPD) of ≥ 5 mm or 4 mm with positive BOP assessed at first re-evaluation following step 2 treatment. The exclusion criteria comprised individuals with unregulated T2DM with an HbA1c scores > 7.5%, other chronic conditions such as rheumatoid arthritis, or pregnancy and lactating. The allocation of sites for therapy per patient was not constrained, and there were no restrictions on the location of residual sites. Both, single- and multi-rooted teeth were considered for the study.

### Sample size calculation

At setting the protocol for the study, no numbers for the proposed therapy option were available from literature so far. Conceptualization of a pilot clinical investigation estimated a relevant average difference in CAL of 1 mm between both study groups with a standard deviation of 1 mm. Thus, the number of necessary cases to demonstrate the anticipated effect with a power of at least 80% was estimated with 24 per group. Accounting for possible dropouts during the study period, the number of patients was increased to 27 in each group.

### Randomization and blinding

Fifty-two patients were randomized into two treatment groups. A computer-generated randomization table was created. Patients were assigned unique numbers from 1 to 52, and 2 sets of randomized numbers were generated (26 for control group subjects and 26 for test). Allocation concealment was performed using sealed envelopes to be opened before SPT treatment after the probing depth was re-evaluated. The generation of the random sequence allocation and the assignment of participants to interventions were performed by the investigator, who performed the treatment herself (L.B.). Thus, there was no blinding of the investigator possible in study setting.

### Treatment protocol

One calibrated investigator (L.B.) performed the subgingival instrumentation under local anesthesia with 4% articaine (Ultracain DS, Sanofi, Germany). The calibration was achieved by measuring the PPD and Recession at the phantom model for diagnosing periodontitis (A-PB Frasaco, Germany) in three separate cycles. The study coordinator (A.F.) controlled the evaluated numbers, calibration fulfilled the requirements since overall agreement between three assessments exceeded 90% level.

Patients enrolled have been re-instructed in oral hygiene measures and treated by the operator (L.B.). The approximal plaque index (API) documented the progress in improving adequate oral hygiene performed by the patients at home [21]. The change in the scores of Approximal Plaque Index (API) compared the level assessed at evaluation after initial therapy with the scores assessed during the study.

The mechanical treatment regimen was identical for sites from both the test and control groups, respectively. In brief, sites underwent subgingival instrumentation by using Gracey curettes (HU-Friedy Group, Chicago, USA), ultrasonic instruments (SONICflex; KaVo, Biberach an der Riß, Germany), and glycine powder air polishing (Airflow; EMS, Vallée de Joux, Switzerland). In the test group (group A), each site intended to treat received subgingivally applied sodium hypochlorite cleaning gel (Perisolv; Regedent AG, Zürich, Switzerland) for 30 s before starting SI. Thereafter, a thorough mechanical debridement of the biofilm followed. The application of sodium hypochlorite gel repeated twice to enhance decontamination effect. An explorer probe (EXS3A6, HU-Friedy Group, Chicago, USA) verified the desired and appropriate instrumentation result. Concomitantly, the sites received 0.2–0.3 ml of xHyA (hyaDENT BG, Regedent AG, Zürich, Switzerland) up to the gingival margin.

Patients received instructions to continue oral hygiene without any restriction after completing the SPT visit regardless treatment group allocation. Subsequently, within the following 7 days, a second application of xHyA (0.2–0.3 ml) was placed subgingivally alongside with the control of oral hygiene. At three months re-evaluation, the treatment was repeated according to patient’s allocation in sites presenting with persistently constant PPD ≥ 5 mm and positive BOP. The final re-evaluation took place 9 months post-treatment (Fig. 1).

### Statistical analysis

Descriptive statistics were applied for the metrical variables pocket probing depth (PPD), clinical attachment level (CAL), and recession, including mean, standard deviation, median, minimum, and maximum. For nominal data (BOP), percentages were calculated to summarize the data. The primary parameter was CAL, while PPD, BOP and Rec applied as secondary parameters. To determine univariate differences between groups Analysis of Variance (ANOVA) or Chi-Square-Test in the case of nominal data were calculated. For the analysis of group effects repeated-measures ANOVA models were performed on the outcomes CAL, PPD, GR. In case of variance heterogeneity across time determined by Mauchly's test of sphericity, Greenhouse–Geisser adjusted testing was applied. Treatment group by time interaction effects were additionally quantified using 95% confidence intervals and partial eta square as effect size η^2^. Values of 0.01–0.06, 0.06–0.14, and more than 0.14 were interpreted as small, medium, or large effects, respectively. Statistical analysis performed with the IBM SPSS 27 software package (IBM Corp.).

Outcomes displayed using estimated marginal means across the time points for both groups. For all analyses, a two-tailed significance level of α = 5% was applied.

## Results

**Fig. 2:**
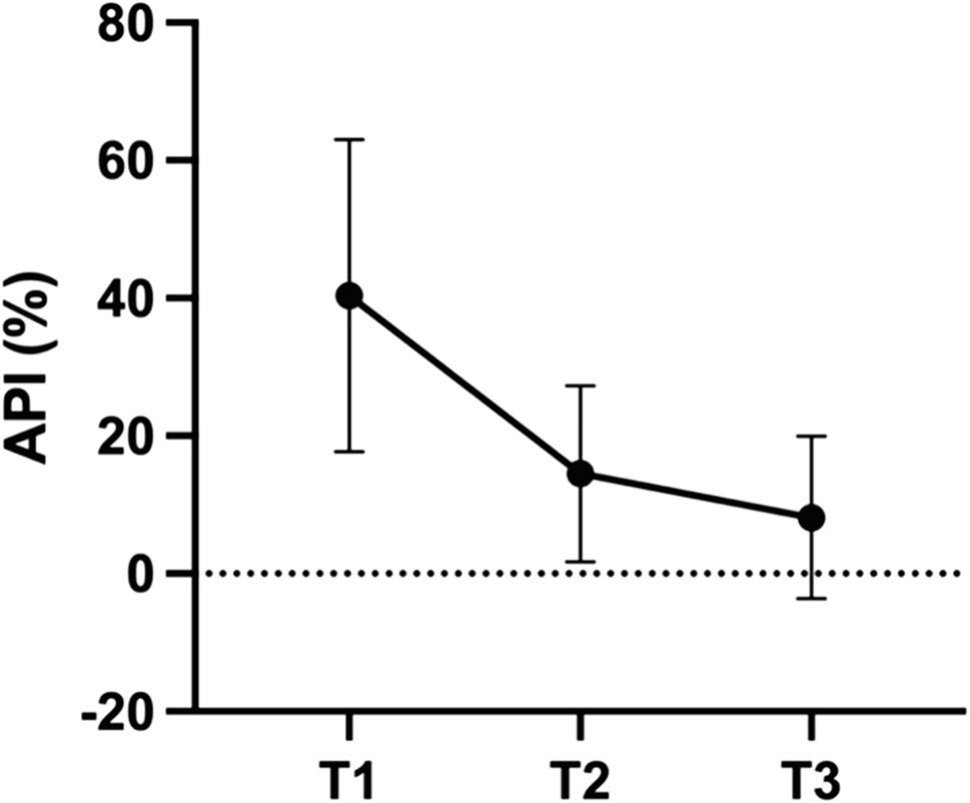
Development of API throughout the observation period

The present study enrolled 52 patients with 1448 sites intended to treat after two patients allocated to group A withdrawn participation agreement. The mean age was 58.6 ± 12.4, with 20 patients being females (40%) and 30 males (60%). All patients were normoglycemic, and 15 patients (30%) were smokers. The oral hygiene improved continuously from the baseline level to the final examination 9 months after starting this trial. Figure 2 displays the positive development of oral hygiene performance at mean level for both groups. Both groups exhibited homogeneity in terms of age, gender, initial number of sites (31.38 ± 21.35 vs. 28.63 ± 17.60, respectively; *p* = 0.622), and BOP frequency at baseline evaluation (Table 1). According to the randomized allocation, 764 sites received the adjunctive protocol (treatment group A), while 684 sites received the subgingival treatment without adjunctives (treatment group B). Summarizing the treated sites by patient allocation, 25 patients underwent treatment by option A and 27 patients by option B. Fifty out of 52 patients completed the study; two dropouts occurred in group B following the 3-month reevaluation. One patient relocated, while the other one had to be excluded due to inadequate compliance. Healing was uneventful in all patients; participants did not report any unexpected adverse events, and the investigator observed neither.

**Table 1:** Demographic data from two groups of patients with controlled homogeneity

	Group A (*n* = 25)	Group B (*n* = 25)	Total (*N* = 50)	*p*-value
Age				
Mean ± SD Median Min Max	60.6 ± 11.3 58 41 82	56.4 ± 13.5 55.5 29 82	58.6 ± 12.4 56.5 29 82	0.232
Gender				
Male Female	19 (73.1%) 7 (26.9%)	11 (45.8%) 13 (54.2%)	30 (60.0%) 20 (40.0%)	0.082
Smoker				
Yes No	7 (26.9%) 19 (73.1%)	8 (33.3%) 16 (66.7%)	15 (30.0%) 35 (70.0%)	0.760
Number of measuring points				
Mean ± SD Median Min Max	31.38 ± 21.35 27.5 5 81	28.63 ± 17.60 25.5 8 82	30.06 ± 19.49 26.0 5 82	0.622

At the outset (T1), baseline mean values for the primary parameter, CAL indicated no significant variance between the two groups (5.85 ± 1.42 mm for group A vs. 6.07 ± 1.59 mm for group B), (*p* = 0.105). Nevertheless, as the intervention effect unfolded, Group A consistently demonstrated a remarkable enhancement in CAL compared to Group B at both 3- and 9-month visits (T2: 4.44 ± 1.37 mm and T3: 3.76 ± 1.18 mm for group A vs. T2: 4.85 ± 1.66 mm and T3: 4.59 ± 1.70 mm for group B, respectively), with these distinctions holding statistical significance (*p* = 0.001).

Similarly, the means for PPD showed no statistically significant divergence between Group A and Group B (T1: 4.74 ± 0.99 mm group A vs. 4.69 ± 1.01 mm group B) (*p* = 0.193, Table 2). As the study progressed, both groups exhibited a notable and significant reduction in PPD (T2: 3.50 ± 1.03 mm in group A and 3.35 ± 1.08 mm in group B; T3: 2.94 ± 0.82 mm and 3.14 ± 1.01 mm, respectively) (Table 2). However, Group A exhibited a more substantial reduction than Group B at both, the 3- and 9-month evaluations and these disparities were statistically significant at the 9-month evaluation (*p* = 0.001, Table 4).

**Table 2:** Alterations in clinical parameters between BL (T1), 3- (T2) and 9-month (T3) visit

	Group A			Group B			*p*-value; partial η^2^
	T1	T2	T3	T1	T2	T3	
GR							
Mean ± SD Median Minimum Maximum	1.12 ± 0.95 1.00 0 5	0.95 ± 0.88 1.00 0 5	0.81 ± 0.82 1.00 0 5	1.38 ± 1.14 1.00 0 5	1.51 ± 1.15 1.00 0 5	1.48 ± 1.15 1.00 0 5	< 0.001; 0.059
PPD							
Mean ± SD Median Minimum Maximum	4.74 ± 0.99 4.00 2 12	3.50 ± 1.03 3.00 1 6	2.94 ± 0.82 3.00 1 5	4.69 ± 1.01 4.00 4 10	3.35 ± 1.08 3.00 1 9	3.14 ± 1.01 3.00 1 8	< 0.001; 0.014
CAL							
Mean ± SD Median Minimum Maximum	5.85 ± 1.42 6.00 4 13	4.44 ± 1.37 4.00 1 9	3.76 ± 1.18 4.00 1 9	6.07 ± 1.59 6.00 4 14	4.85 ± 1.66 5.00 2 12	4.59 ± 1.70 4.00 1 11	< 0.001; 0.031
BOP							
Mean ± SD Median Minimum Maximum	0.78 ± 0.42 1.00 0 1	0.17 ± 0.48 0.00 0 9	0.11 ± 0.32 0.00 0 1	0.78 ± 0.42 1.00 0 1	0.21 ± 0.41 0.00 0 1	0.13 ± 0.34 0.00 0 1	0.360; 0.001

**Fig. 3:**
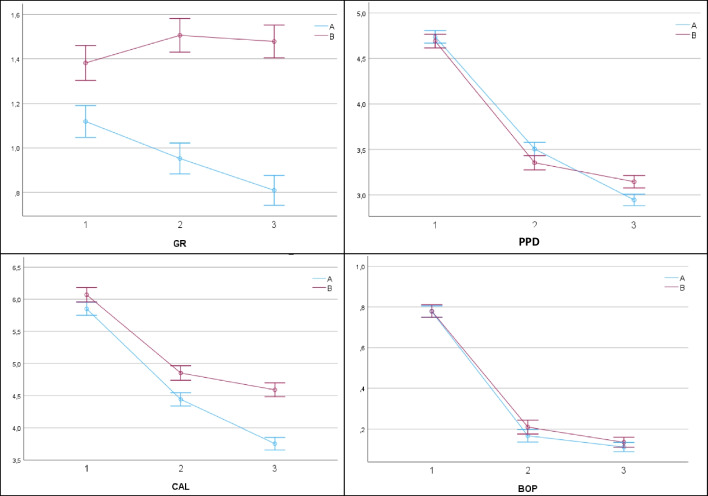
**a-d** Clinical parameters and their alterations during 9-month observation, estimated Marginal Means and their 95% confidence intervals as computed by the General Linear Model

Gingival Recession (GR) scores were significantly different between Group A and B at baseline. Throughout the 9-month evaluation, Group A displayed insignificant but rather positive changes, while Group B showed little progression in GR depth from Baseline to 3 months, remaining constantly at the same level for a further 6 months. The intergroup difference in mean GR level at both evaluations was statistically significant (*p* = 0.001) (Fig. 3a-d).

Conversely, the BOP frequency yielded no statistically significant difference between the two groups at any time point, neither at baseline, at 3-months, nor 9-month evaluation, with *p*-values of 0.796, 0.175, and 0.339, respectively. Details of the clinical parameters and their alterations presented in Table 2. The frequency of pocket closure (PPD ≤ 4 mm / BOP negative) after the 9-month observation period was indifferent in the shallow pockets (4-5 mm). Interestingly, the pocket closure rate decreased in the deeper pockets but was still higher in the treatment group. In the very deep pockets > 7 mm, group A exhibited a pocket closure rate of 94%, while the control group only exhibited 42% pocket closure (Table 3).

**Table 3:** Pocket closure effect per treatment group based on residual PPD ≤ 4 mm and negative BOP at 9-month reevaluation stratified by initial PPD at patient enrolment

BL PPD (mm)	N sites treated per subgroup	N sites treated per allocation to A or B	N sites applying to pocket closure	Rate of pocket closure effect
Severe ≥ 8	**36**	**17** sites group A	**16**	94%
		**19** sites group B	**8**	42%
Moderate = 6–7	**214**	**126** sites group A	**93**	73%
		**88** sites group B	**58**	66%
Shallow = 4–5	**1248**	**671** sites group A	**633**	94%
		**577** sites group B	**521**	90%

The inter-group comparison of the means for all four parameters at any time point of the study displayed by Table 4, showing the respective level of significance and the partial effect size (**partial η**^**2**^).

**Table 4:** Between group effects for the different time points

	Group A vs. Group B		
	T1	T2	T3
GR			
*p*-Value partial η^2^	< 0.001 0.009	< 0.001 0.057	< 0.001 0.090
PPD			
*p*-Value partial η^2^	0.193 0.001	< 0.001 0.010	< 0.001 0.009
CAL			
*p*-Value partial η^2^	0.105 0.002	< 0.001 0.009	< 0.001 0.066
BOP			
*p*-Value partial η^2^	0.796 < 0.001	0.175 0.001	0.339 0.001

## Discussion

The present study evaluated the clinical effect of the adjunctive combination of sodium hypochlorite/amino acid gel and xHyA for treating residual inflamed periodontal pockets during SPT in a private praxis environment. Clinically, the treatment and the healing were uneventful in all cases. The study results reveal that repeated instrumentation during SPT was an effective option to further improve clinical periodontal parameters in both groups. However, the adjunctive protocol investigated in this study yielded better results than the control group regarding attachment level gain. Intriguingly, the pocket closure rates were significantly higher in test group as compared to the controls when looking at deep residual pockets (Table 3), indicating that the adjunctive protocol was highly beneficial for deep residual sites. Thus, the response in sites designated for surgical intervention due to scores assessed at re-evaluation emphasized the probability of waiving surgery by means of meticulous instrumentation supported by adjunctive use of hypochlorite/amino acids and xHyA.

The three-months evaluation of periodontal conditions after completion of initial periodontal treatment applies to German regulations for treating periodontitis within the health insurance system, but is also backed up by the recent meta-analysis [22]. The data extracted from 29 RCT studies leave no room for the assumption the re-evaluation performed three months after completion of initial subgingival instrumentation may impair ongoing healing. While our group set up the protocol and conducted the clinical part for the step 3 SPT treatment in a private praxis environment, the group from Lithuania used the same adjunctive protocol at step 2 treatment of periodontal pockets [23]. The authors reported significantly better PD reduction over an observation period of 6 months for the test group compared to controls (2.9 ± 0.4 vs 1.8 ± 0.6 mm, *p* < 0.001, respectively). Similarly, mean CAL gain was statistically higher in the test group compared to the control one (test: 2.6 ± 0.5 vs. control: 1.6 ± 0.6 mm, *p* < 0.001). The notable pocket closure rate in sites with PPD exceeding 6 mm dropped from total of 298 (8.7%) to 4 (0.1%) in test group (p = 0.003), and from 277 (7.6%) to 35 (1.0%) in the controls. The declining detection rates for five targeted periodontal pathogens in samples obtained from subgingival compartment at three clinical visits accordingly to clinical examinations reflected the abovementioned benefit of the adjunctive protocol applied at step 2 periodontal therapy. The detection frequencies for *A.actinomycetemcomitans*, *P. gingivalis*, *Pr. Intermedia*, *T. denticola* and *T. forsythsia* were significantly smaller in the test group vs. controls in 6-month samples [24]. Thus, the counts of the targeted putative periodontal pathogens showed significantly and continuously reduced scores in sites that showed the greatest clinical response in the test group, whereas the controls demonstrated a kind of rebound in colonization by the same microorganisms during the observation period of 6 months. The conformity of clinical and microbiological outcomes underscored the benefit of the combined hypochlorite/ amino acid gel use followed by xHyA application after completed NSPT. Although this study addressed another step in the treatment flow of periodontal pockets, our clinical outcome agreed with the data cited above pointing out the universal benefit proposed approach unfolds in a non-surgical treatment for deep periodontal pockets. The clinical parameters, PPD, CAL, BOP improved significantly from baseline to 6-months reevaluation in a case series which used same adjunctives during step 2 therapy [23].

Similar clinical results we recorded in this study over a period of 9 months post-op. However, the treatment population was recruited from the patients who presented with residual active periodontal pockets and were referred to either step 3 or 4 – the SPT. Intriguingly, the pocket closure rates were substantially higher in the treatment group, while patients with shallow pockets did not seem to benefit from the adjunctive treatment. This observation may partly explain the contradicting results for xHya-supported surgical and non-surgical treatment protocols [25]. A very similar rate in BOP frequency in both groups consistently recorded from baseline to latest re-evaluation may be related to the masking effect which smoking induces on bleeding on probing frequency by duration of the habit [26]. In our study, about 30% of participants in both groups were smokers.

In vitro studies, sodium hypochlorite has proven effective against gram-negative species-dominated biofilms [27]. In addition, coating dentin surfaces with hypochlorite/amino acid also promoted the proliferation of periodontal ligament fibroblasts on these surfaces in vitro [28]. Moreover, subgingivally applied as a singular adjunctive for treating periodontitis, the hypochlorite/amino acid gel has been shown to enhance antimicrobial and therapeutic effects expressed by improved clinical parameters in randomized clinical studies [29, 30]. However, in a randomized clinical trial, the additional use of sodium hypochlorite gel did not affect the outcomes of manual or ultrasonic subgingival instrumentation in a limited group of patients at the SPT stage [31].

Hyaluronic acid, a ubiquitous component in mammalian tissues, exhibits a versatile spectrum of properties. Studies have demonstrated that HA has a bacteriostatic effect, contributing to minimizing bacterial contamination in surgical wounds [32]. Additionally, HA has a fungistatic effect on the growth of Candida albicans [33]. However, using xHyA as an adjunct to mechanical treatment of periodontal pockets failed to unleash a greater effect than subgingival instrumentation alone in a 12-month randomized non-surgical study [14].

Results from animal studies with a surgical approach proved the potential of xHyA to support new attachment formation in a recession canine model, an acute intrabony defect, and an acute furcation Grade 3 model. The microscopic outcome in all these experiments proved new cementum, ligamentum, and bone formation, respectively. Histomorphometric assessment revealed a clinically relevant amount of newly organized tissues in sites treated with xHyA [34–36]. HA presence can also promote osteosynthesis by enhancing mesenchymal cell differentiation in critical-sized bone defects and has been shown to stimulate osteoblasts, underscoring a great potential for periodontal regeneration [37, 38].

Accordingly, a randomized controlled trial comparing the efficacy of enamel matrix derivatives and xHya in treating contained three-wall intrabony defects reported equal regenerative outcomes for both biologics [39]. Effective decontamination of the root surface and periodontal pocket is a prerequisite for effective regeneration and represents a major rationale for flap elevation. Same requirement is a particular challenge for achieving regenerative healing by non-surgical approach. The recent animal study performed in chronic infected intrabony pockets by means of non-surgical subgingival instrumentation corroborated at histomorphometrical level the regenerative pattern of tissue formation if the adjunctive hypochlorite/amino acid and xHyA treatment was applied [40].

Naturally, this study exhibits some limitations. Because it is a single-center investigation in a private clinic involving just one investigator, a chance of investigator bias is inherent to this study. Moreover, radiographic validation of the clinical findings is warranted in future studies.

Nonetheless, the study's findings were clinically relevant, advocating for incorporating proposed adjunctive therapy in residual periodontal pocket management. Moreover, the high pocket closure rate of deep sites indicates that the adjunctive use of sodium hypochlorite and xHya may circumvent surgical intervention. Future studies must, therefore employ more independent and blinded investigators to increase the generalizability of findings.

## Conclusions

Within the limitations of this mono centre RCT study we consider the proposed adjunctive treatment protocol sufficient in improving clinical conditions in persisting residual periodontal pockets at relevant level during SPT visit. Thus, patients showing off with residual deep sites at reevaluation may carry a sustained benefit from such extended therapy effort.

## Data Availability

The data that support the findings of this study are not openly available due to reasons of sensitivity and are available from the corresponding author upon reasonable request. Data are located in controlled access data storage at Witten/Herdecke University, Department of Periodontology.
